# 290. Quantitative Antibody Levels Against SARS-CoV-2 Spike Protein in Children with COVID-19 and MIS-C

**DOI:** 10.1093/ofid/ofac492.368

**Published:** 2022-12-15

**Authors:** Gulhadiye Avcu, Sema Yıldırım Arslan, Mehmet Soylu, Melis Bilen, Candan Çiçek, Zümrüt Şahbudak Bal, Nuri Zafer Kurugöl, Ferda Ozkinay

**Affiliations:** Ege university, pediatric infectious diseases, izmir, Izmir, Turkey; Division of Infectious Disease, Department of Pediatrics, BORNOVA, Izmir, Turkey; Ege university, medical microbiology, izmir, Izmir, Turkey; Ege university, izmir, Izmir, Turkey; Ege University School of Medicine Department of Microbiyology, İzmir, Izmir, Turkey; Division of Infectious Disease, Department of Pediatrics, BORNOVA, Izmir, Turkey; Division of Infectious Disease, Department of Pediatrics, BORNOVA, Izmir, Turkey; Ege university, izmir, Izmir, Turkey

## Abstract

**Background:**

Severe acute respiratory syndrome coronavirus 2 (SARS-CoV-2) has different clinical courses in children and adults. The majority of coronavirus diseases 2019 (COVID-19) caused by SARS-CoV-2 are either mild or asymptomatic in children.

This report aimed to determine quantitative antibody levels against SARS-CoV-2 spike protein in children with COVID-19 and MIS-C.

**Methods:**

A single-center retrospective study was conducted at a tertiary-level university hospital. Seventy-five pediatric patients diagnosed with COVID-19 and MIS-C were included between September 2021 and February 2022.

The demographic, clinical and laboratory data of cases were extracted from medical records. The duration of hospitalization, management, and outcomes was reported.

Quantitative antibody levels against SARS-CoV-2 spike protein in the third month after the initial diagnosis was measured, and patients were also evaluated for post-COVID-19 syndrome symptoms .

**Results:**

The patients were categorized into three disease phenotypes; there were 36 (48%) patients with mild/asymptomatic (group1)( M/F 20/16, mean age, 11.4 years) 22 (29.3%) patients with moderate to severe SARS-CoV-2 infection (group 2)(M/F 13/9, mean age, 13 years) and 17 (22.6%) patients with MIS-C (group 3)( M/F 9/8, mean age, 10.1 years).

The majority of the children in group 1 (80.6%), in group 2(90.9%), and in group 3(82.4%) had a detectable IgG antibodies to SARS-CoV-2 spike protein(p=0.567). The mean antibody values against SARS-CoV-2 spike protein was 321.9±411.6 in group 1, 274±261 in group 2, and 220±299 in group 3 (p >0.05).

The antibody positivity rate was similar in patients with COVID-19(85.5%) and MIS-C (82.4%) (p=0.833). The mean antibody value against SARS-CoV-2 spike protein was 303.9±360.3 in the COVID-19 group and 220 ±299 in the MIS-C group(p >0.05).

**Conclusion:**

The majority of the children had a detectable IgG antibody level to SARS-CoV-2 spike protein. There was no difference between asymptomatic/mild disease, moderate/severe disease, and MIS-C groups in mean antibody levels. Long-term studies are needed to examine antibody responses over time in children with COVID-19 and MIS-C for different vaccination schedules.

**Disclosures:**

**All Authors**: No reported disclosures.

